# Superficial Venous-Associated Inflammation from Direct IV Administration of RRx-001 in Rats

**DOI:** 10.7150/ijms.76615

**Published:** 2022-09-21

**Authors:** Scott Caroen, Bryan Oronsky, Tony Reid, Karamjeet Pandher, Alric Lopez

**Affiliations:** 1 EpicentRx Inc., 11099 North Torrey Pines Road Suite 160, La Jolla, CA 92037, USA.; 2 Sinclair Research, 562 State Road DD, Auxvasse, MO 65231, USA.

**Keywords:** Intravenous infusion, phlebitis, small molecule, RRx-001, drug-device combination

## Abstract

RRx-001 is a small molecule NLRP3 inflammasome inhibitor with anti-CD47 and antiangiogenic/vascular normalization properties in a Phase 3 clinical trial that has been designated as a drug-device combination by the FDA. In the Phase 1 first-in-man dose escalation clinical trial, where RRx-001 was given by direct intravenous (IV) infusion, the main adverse event was a sterile painful infusion phlebitis (IP). Less pain was experienced when RRx-001 was infused at a slower rate over multiple hours which was impractical on an outpatient basis. In Phase 2, for reasons of convenience and safety, RRx-001 was co-administered with an aliquot of autologous blood from an *ex-vivo* device called the eLOOP on the premise that RRx-001 binds to hemoglobin on red blood cells (RBCs), making it unavailable to directly interact with venous nociceptors. Phlebitis has the potential to progress to deep venous thrombosis or septic thrombophlebitis or post-thrombotic syndrome in hypercoagulable and immunosuppressed cancer patients. In this 13-week toxicology study of once weekly IV RRx-001 administration to Wistar Han rats followed by a recovery period of 28 days. The main observed toxicity was a significant inflammatory response in the vein wall, consistent with superficial venous thrombosis observed in man. Due to this development, direct IV infusion of RRx-001 is relatively contraindicated in favor of co-administration with autologous blood.

## Introduction

RRx-001 is a small molecule NLRP3 inflammasome inhibitor 1 with anti-CD47 2 and antiangiogenic/vascular normalizing 3 properties in a Phase 3 clinical trial 4 that has been designated as a drug-device combination by the FDA. In the Phase 1 first-in-man dose escalation clinical trial, where RRx-001 was given by direct intravenous (IV) infusion in doses that ranged from 20-166 mg (10-83 mg/m^2^), the main adverse event was a painful sterile infusion phlebitis (IP), a clinical diagnosis, characterized by tender, dilated and inflamed superficial veins during administration in part from nitric oxide (NO) release 5. Pain (but not necessarily phlebitis) was ameliorated with RRx-001 infusion over multiple hours, which is unduly burdensome and time-consuming to perform on an outpatient basis.

In subsequent Phase 2 studies, for reasons of convenience and safety, RRx-001 was co-administered over 10 minutes with an aliquot of autologous blood from an *ex-vivo* device on the premise that binding of RRx-001 to hemoglobin 6 in the device would render it unable to directly elicit venous pain and irritation *in vivo*, which is, in fact, what occurred. Phlebitis has the potential to lead to significant morbidity and mortality in hypercoagulable and immunosuppressed cancer patients from the development of post-thrombotic syndrome, deep venous thrombosis or septic thrombophlebitis.

In this 13-week toxicology study of once weekly direct RRx-001 IV administration to Wistar Han rats followed by a recovery period of 28 days, increased incidence and severity of subacute inflammation and increased incidence of chronic thrombi were observed at the injection site. These findings are consistent with superficial venous thrombosis, as observed in man.

## Materials and Methods

### Animal Model and Protocol

One hundred and forty animals were assigned to four male or female groups and were administered the vehicle or test article at dose levels of 0, 0.25, 4, or 4/12 mg/kg/dose RRx-001, once weekly for 13 weeks via a 10-minute infusion using a syringe pump and a femoral vein indwelling catheter, or the tail vein (when indwelling catheter became non-functional). Five male or female animals given 0 or 4/12 mg/kg were assigned to a test article-free recovery phase of 28 days. Detailed experimental design information can be found in Table 1.

Clinical pathology samples were collected and analyzed. At scheduled terminations (end of dosing phase and end of recovery phase), all surviving animals were euthanized. Protocol-specified tissues were collected from each animal for pathology evaluation. All samples were preserved in appropriate fixative and sent to Histo-Scientific Research Laboratories [a StageBio company] for histology processing. The samples were trimmed, embedded in paraffin, sectioned and stained with hematoxylin and eosin (H&E). The resulting slides were sent to the Test Facility for pathology evaluation of Vehicle Control and High Dose Main sacrifice animals. Microscopic findings were scored on scale of 0 to 5 as follows: 0 = No Finding, 1 = Minimal, 2 = Mild, 3 = Moderate, 4 = Marked, and 5=Severe.

### Histopathology

All protocol specified tissues from control and high-dose animals were comprehensively examined by an American College of Veterinary Pathologists (ACVP)-certified pathologist. The pathology findings were peer-reviewed by an ACVP-certified pathologist.

## Results

At the end of the dosing phase, minimal venous changes and few inflammatory cells were present in the control groups. In mid and high RRx-001-treated groups, at the dosing site, increased incidence and severity of subacute inflammation and increased incidence of chronic thrombi were present. Subacute inflammation was characterized by loss of endothelium, fibrin thrombi, fibrin in the vessel wall and surrounding connective tissue, presence of inflammatory cells, neutrophils and mononuclear cells, in the vessel wall and surrounding connective tissue, and hemorrhage. The presence of recanalized thrombi is consistent with regression and resolution of the thrombi.

At the end of the non-dosing recovery phase (28 days), RRx-001-related microscopic findings were only present at the dose site in rats administered the high dose of RRx-001. These findings, which included minimal increased pigment, minimal thickening of the tunica media, and/or presence of recanalized thrombi, represent resolution of subacute inflammation observed at the end of the dosing phase.

## Conclusion

This toxicology study demonstrates an association between direct RRx-001 IV administration and venous thrombosis and inflammation of the vein wall in part from NO release, which is also seen in the Phase 1 first-in-man study. The recommended/optimal method of RRx-001 administration is via mixture with blood, which almost completely eliminates the occurrence of venous phlebitis in humans, unlike the endothelial irritation/injury and inflammatory activation from direct IV infusion. Traditionally, superficial venous thrombophlebitis 7 is considered to be a benign and self-limiting complication; however, more severe sequelae are possible especially in cancer patients i.e., deep venous thrombosis, septic thrombophlebitis or post-thrombotic syndrome 8 whose clinical manifestations may include pain, edema, erythema, new varicose veins, hyperpigmentation, skin thickening and, in severe cases, ulcers; hence, for these reasons, direct IV infusion with RRx-001 is relatively contraindicated in favor of co-administration with an aliquot of autologous blood in an *ex-vivo* device called the eLOOP. Hence, on this basis, for reasons of safety, RRx-001 is designated by the FDA as a drug-device combination.

## Figures and Tables

**Table 1:** Experimental Design

Group	Treatment	Number of Animals		Dose Conc.^C^ (mg/mL)	Dose Volume (mL/Kg)	Dose Level^C^ (mg/Kg)
		Main^A^	Recovery^B^			
1	Vehicle Control	15 M/15 F	5 M/5 F	0	5	0
2	Low Dose	15 M/15 F	-	0.05	5	0.25
3	Mid Dose	15 M/15 F	-	0.8	5	4
4	High Dose	15 M/15 F	5 M/5 F	0.8/2.4	5	4/12

^A^ Main study animals were necropsied on Dosing Phase Day 86. ^B^ Recovery study animals were necropsied on Recovery Phase Day 114. ^C^ Animals in Group 4 received a dose of 4 mg/kg on Dosing Phase Days 1 and 4 and then doses of 12 mg/kg for the remainder of the study. Animals in Group 1 also received a dose of vehicle on Dosing Phase Day 4.
